# Doxorubicin promotes the production of inflammatory cytokines in tumor-associated macrophages through activating lactate dehydrogenase A

**DOI:** 10.1038/s41420-026-03014-0

**Published:** 2026-03-31

**Authors:** Bin Liu, Wei Yang, Shuo Feng, Xingning Jiang, Jingfan Lu, Fei Wang, Yaowen Hu, Yanhao Liu, Haifeng Ma, Ting Sun

**Affiliations:** 1 Neurosurgery and Brain and Nerve Research Laboratory, First Affiliated Hospital of Soochow University, Suzhou, China; 2 Department of Neurosurgery, Qinghai Provincial People’s Hospital, Xining, China; 3 State Key Laboratory of Radiation Medicine and Protection, School of Radiation Medicine and Protection and Collaborative Innovation Center of Radiation Medicine of Jiangsu Higher Education Institutions, Soochow University, Suzhou, China

**Keywords:** Cancer metabolism, Immunoediting, CNS cancer

## Abstract

Glioblastoma (GBM) presents a significant challenge because of its immunosuppressive microenvironment. The standard treatment protocol, including surgery, radiotherapy, and temozolomide, has been unable to alleviate immunosuppression. Doxorubicin chemotherapy induces immunogenic cell death in cancer cells, reshaping an immune-activated microenvironment. Here, we investigated the mechanism of immune activation induced by doxorubicin in tumor-associated macrophages (TAMs). Radiotherapy and temozolomide plus doxorubicin inhibited tumor growth and reduced the levels of immunosuppressive markers. Mechanically, doxorubicin promotes the production of lactate through activating lactate dehydrogenase A (LDHA) to upregulate the transcription of inflammatory cytokines. Our study confirmed a new mechanism by which doxorubicin remodels the tumor microenvironment by promoting the glycolytic process and lactic acid production, suggesting that combining radiotherapy and temozolomide with doxorubicin chemotherapy may be a potential strategy for GBM treatment.

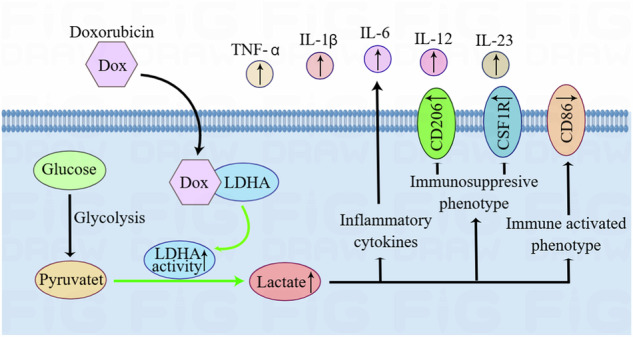

## Introduction

Glioblastoma (GBM) is a highly heterogeneous, aggressively invasive, and very malignant tumor located in the central nervous system. Although treatments, such as radiotherapy (RT), chemotherapy, and targeted therapies have improved, patient prognosis remains poor [1]. Studies have shown that the interactions between GBM and the tumor microenvironment (TME) play a role in immunosuppression within GBM [2].

Within the complex microenvironment of GBM, myeloid cells are vital players in tumor-related immune responses and are involved in various processes, such as tumor initiation, progression, immune evasion, and treatment resistance. The myeloid cells that infiltrate GBM are known as tumor-associated macrophages (TAMs) [3]. TAMs represent the most abundant myeloid cell population in the GBM microenvironment and are generally categorized into pro-inflammatory M1 and anti-inflammatory M2 subtypes. These TAMs not only support the growth and progression of GBM but contribute to its resistance to therapies [4].

Macrophages undergo notable metabolic alterations during their polarization, which affect their function [5]. Inflammatory macrophages that are classically activated exhibit increased glycolysis alongside reduced oxidative phosphorylation [6]. This metabolic shift toward glycolysis is a key feature of inflammatory macrophages. The role of glycolysis in the activity of these cells is complex, and metabolic interactions play a vital role in finely tuning the activation of innate immune cells [7]. The current understanding proposes that distinct metabolic pathways are engaged at various stages to boost the functions of inflammatory macrophages, resulting in the expression of proinflammatory genes [8].

Doxorubicin, an inhibitor of topoisomerase II, induces DNA strand breaks and interferes with the activity of topoisomerases that are essential for DNA replication and transcription [9]. It has demonstrated significant therapeutic efficacy and is recognized as a potent chemotherapy agent approved by the Food and Drug Administration (FDA) for the treatment of various cancers, including carcinomas, breast cancer, and hematological malignancies, among others [10]. Moreover, doxorubicin can increase the infiltration of CD8 + T cells and natural killer (NK) cells, as well as stimulate pro-inflammatory activation of macrophages and dendritic cells [11]. Nonetheless, the exact mechanism through which doxorubicin enhances immune activation remains unclear.

Here, we explored the molecular mechanism by which doxorubicin enhances the efficacy of RT and temozolomide (TMZ) chemotherapy for GBM. It was found that doxorubicin enhances the anti-tumor immune response by inducing the inflammatory activation of TAMs. The role of glucose metabolism in the activation of TAMs induced by doxorubicin was investigated, and our data showed that the increase in lactic acid produced by glycolysis may be one of the key mechanisms for the inflammatory activation of TAMs.

## Results

### Doxorubicin enhanced the effect of RT and TMZ while unchanged the proportion of myeloid cells in the TME

**Fig. 1:**
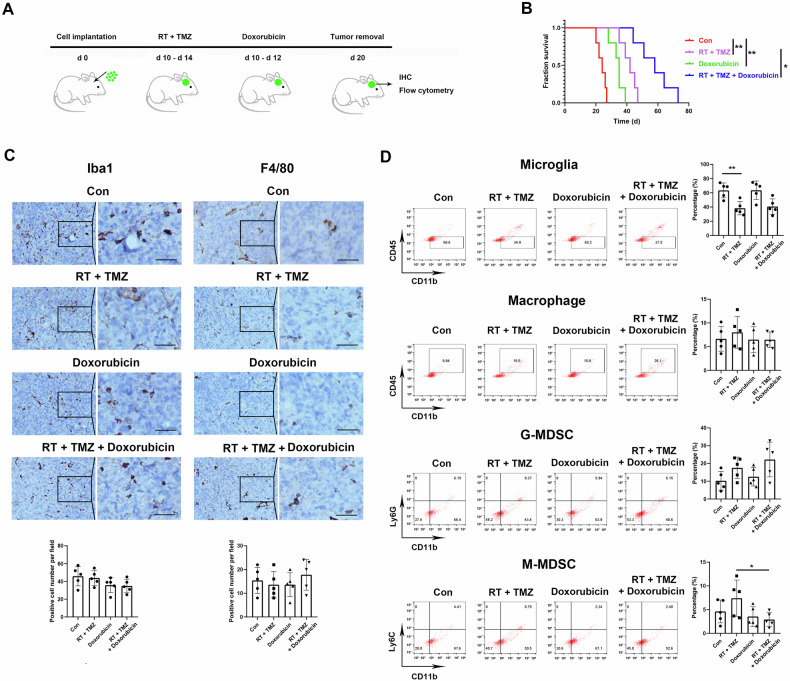
Doxorubicin administration unchanged the proportions of myeloid subpopulations in the TME of the GBM mouse model. C57BL/6 J mice with orthotopic GL261 cell grafts were locally irradiated and administered orally TMZ plus doxorubicin. **A** Treatment plan diagram for the GBM mice. **B** Survival of tumor-bearing mice. **C**,**D** Tumor tissues in the mouse brain were removed on day 20 for IHC staining and flow cytometry detection. **C** Slices from tumor tissues were stained with Iba1 and F4/80 antibodies, and images were captured with a light microscope. **D** Tumor tissues were dissociated, and flow cytometry was employed for the analysis of myeloid subpopulations. The percentages of all subpopulations were analyzed in CD45+ cells. Scale bars correspond to 50 μm. *N* = 5. *P < 0.05, **P < 0.01.

Doxorubicin was widely distributed in tumor tissues of the mice (Fig. S1). Doxorubicin was administered into a GL261 cell-implanted mouse model in combination with RT and TMZ therapy (Fig. 1A). Survival analysis showed that doxorubicin significantly extended the survival time of GBM mice compared with the control. Meanwhile, the combination of doxorubicin, RT, and TMZ consistently enhanced the survival of mice compared with RT and TMZ (Fig. 1B).

To investigate whether doxorubicin enhanced the anti-tumor effect of RT and TMZ by the infiltration of myeloid cells in the TME, we analyzed the percentage of myeloid cells using IHC staining and flow cytometry. The results of both IHC (Fig. 1C) and flow cytometry (Fig. 1D) showed no significant differences in the percentages of microglia, macrophages, granulocytic myeloid-derived suppressor cells (G-MDSCs), and monocytic MDSCs (M-MDSCs) following doxorubicin administration.

### Doxorubicin inhibited M2-like polarization of TAMs

**Fig. 2:**
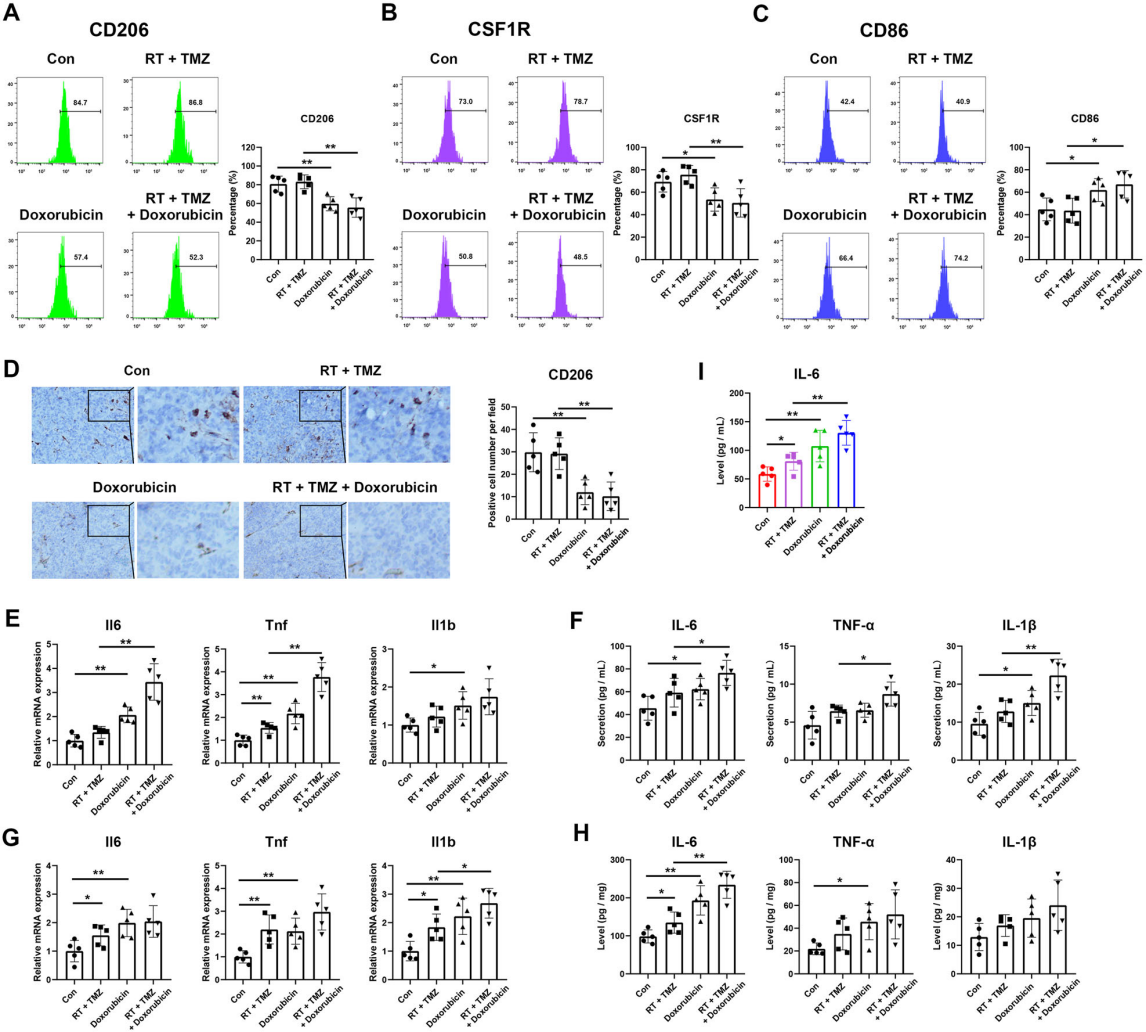
Doxorubicin decreased immunosuppression and increased immune activation of TAMs. The levels of CD206 (**A**), CSF1R (**B**), and CD86 (**C**) in CD11b+ cells were detected using flow cytometry. **D** IHC staining showed CD206 positive cells. Scale bars correspond to 50 μm. **E** The mRNA expressions of cytokines in BMDMs of tumor-bearing mice. **F** The protein levels of cytokines in the supernatants of BMDMs of tumor-bearing mice. **G** The mRNA expressions of cytokines in tumor tissues of the mice. **H** The protein levels of cytokines in tumor tissues of the mice. **I** IL-6 level in the serum of tumor-bearing mice. *N* = 5. *P < 0.05, **P < 0.01.

We examined the status of TAMs changed by doxorubicin administration in the TME. The analyses using flow cytometry demonstrated that doxorubicin decreased the level of the M2-like polarization marker CD206 (Fig. 2A) and the immunosuppressive marker CSF1R (Fig. 2B) and increased the level of the immune activation marker CD86 (Fig. 2C), whether combined with RT and TMZ or not. IHC staining consistently showed a reduced CD206 expression due to doxorubicin administration (Fig. 2D). These results suggested that doxorubicin inhibited M2-like polarization and promoted pro-inflammatory activation of TAMs in the TME of GBM mice.

### Doxorubicin enhanced the inflammatory response in the TME

When macrophages are stimulated by inflammation, they secrete cytokines, such as tumor necrosis factor (TNF)-α, interleukin (IL)-1β, IL-6, IL-12, and IL-23 [12]. To explore doxorubicin-mediated inflammatory responses in vivo, bone marrow-derived macrophages (BMDMs) were isolated after the tumor-bearing mice were administered RT and TMZ in combination with doxorubicin, and the transcription of inflammatory cytokines was assessed. The mRNA expressions of Il6, Tnf, and Il1b were significantly higher in BMDMs after doxorubicin administration than in the control (Fig. 2E). The secretion of these cytokines was also elevated in the supernatants in the groups receiving the RT and TMZ combination plus doxorubicin compared with RT and TMZ group (Fig. 2F). Furthermore, the mRNA and protein levels of these cytokines were consistently upregulated in tumor tissues of the mice after doxorubicin administration (Fig. 2G, I). In addition, increased concentrations of IL-6 in mouse serum were found after doxorubicin administration (Fig. 2I), whereas TNF-α and IL-1β production was undetected (data not shown), suggesting that doxorubicin upregulates IL-6, TNF-α, and IL-1β expression in vivo.

### Doxorubicin enhances the production of inflammatory cytokines in vitro

**Fig. 3:**
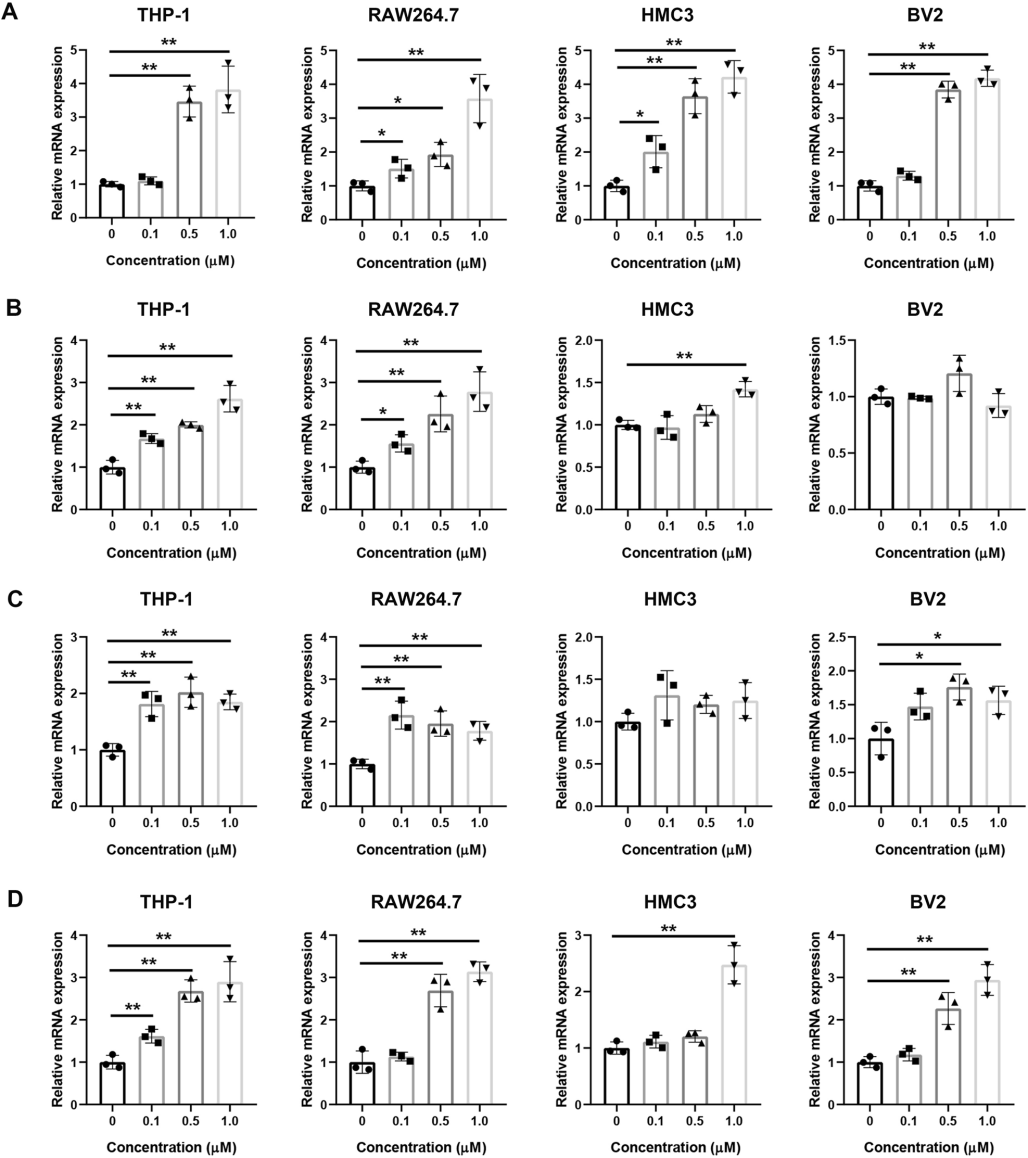
Doxorubicin elevates the transcription of inflammatory cytokines. Macrophages and microglia were treated with different concentrations of doxorubicin for 24 h, then mRNA was extracted and examined using real-time RT-PCR. The relative mRNA expressions of IL6 (**A**), IL12B (**B**), IL23A (**C**), and IL1B (**D**) in THP-1, RAW264.7, HMC3, and BV2 cells were shown. *N* = 3. *P < 0.05, **P < 0.01.

We next measured the production of inflammatory cytokines in cultured macrophages and microglia in vitro. Less than 1 μM of doxorubicin showed no significant cytotoxicity (Fig. S2). The transcription of IL6 showed a dose-dependent increase after doxorubicin treatment (Fig. 3A). The mRNA expression of IL12B was elevated in THP-1, RAW264.7, and HMC3 cells (Fig. 3B); however, the upregulation of IL23A mRNA expression was confirmed in THP-1, RAW264.7, and BV2 cells (Fig. 3C). The transcription of IL1B also showed a trend toward increasing after doxorubicin treatment (Fig. 3D). Taken together, our results support that doxorubicin increases the expressions of a subset of inflammatory cytokines and promotes inflammatory responses.

### Doxorubicin upregulates inflammatory cytokine transcription by glucose metabolism

**Fig. 4:**
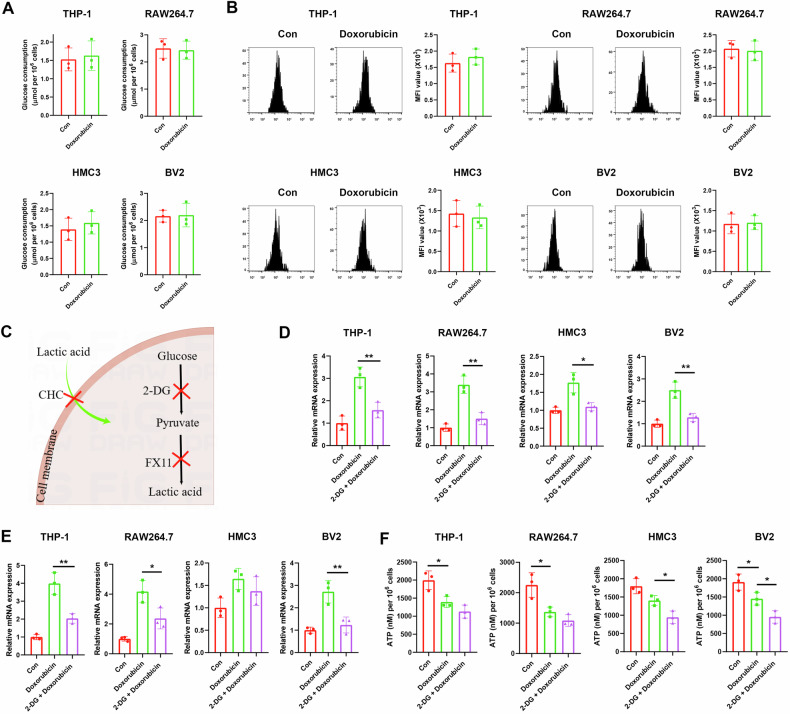
Doxorubicin alters cytokine transcription by glucose metabolism. Macrophages and microglia were treated with 0.5 μM doxorubicin for 24 h. **A** Glucose consumption in the supernatants. **B** Intracellular glucose uptake after 6-NBDG mixture. **C** Diagram of inhibitors in the metabolic processes. Cells were pretreated with 5 mM 2-DG for 0.5 h, the mRNA expressions of inflammatory cytokines TNF (**D**) and IL1B (**E**) and ATP levels (**F**) in cells were assayed. *N* = 3. *P < 0.05, **P < 0.01.

TAMs are highly dependent on specific metabolic reprogramming in response to internal and external stress when performing functions [5]. To address whether the changes in cytokines induced by doxorubicin are mediated by metabolic reprogramming, glucose uptake was examined to analyze the effect of doxorubicin on glucose metabolism. The results showed that glucose consumption and uptake were unchanged after doxorubicin treatment (Fig. 4A, B).

We next treated macrophages and microglia with doxorubicin in the presence of 2-DG, an inhibitor of glucose metabolism (Fig. 4C). Under the conditions we used, 2-DG showed no significant cytotoxicity (Fig. S3). The doxorubicin-induced increase in cytokine transcription was blocked by 2-DG treatment (Fig. 4D, E).

The change in glucose metabolism disrupts the tricarboxylic acid (TCA) cycle fueling [13], due to the interconnection of metabolic pathways. We then examined ATP production. Doxorubicin treatment significantly reduced intracellular ATP levels in THP-1, RAW264.7, and BV2 cells, and 2-DG treatment also lessened ATP levels in HMC3 and BV2 cells after doxorubicin treatment (Fig. 4F), suggesting deceleration of TCA cycle fueling by doxorubicin treatment.

### The effect of doxorubicin was mediated by the LDHA/lactic acid axis

**Fig. 5:**
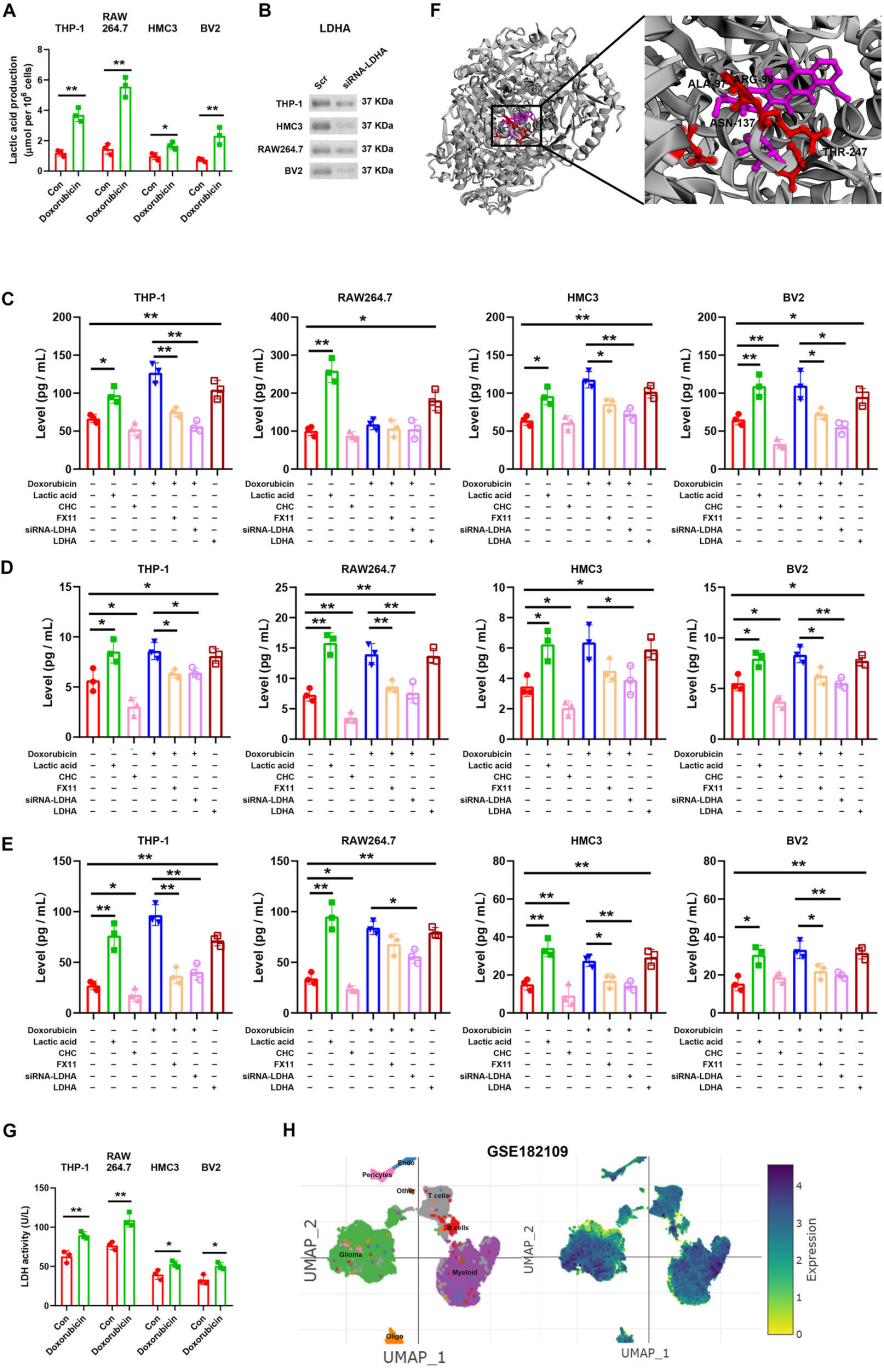
The effect of doxorubicin was induced by lactate. **A** The concentration of lactic acid in the supernatants. **B** Western blotting analysis of LDHA expression after transfection of siRNA targeting LDHA. **C–E** Cells were treated with 100 mM sodium lactate for 0.5 h, or 2 mM CHC for 6 h, or 0.5 μM doxorubicin for 24 h with or without 20 μM FX11 pretreatment for 3 h, or cells were transfected with LDHA-gRNA for gene knockdown, then treated with doxorubicin. The levels of IL-6 (**C**), TNF-α (**D**), and IL-1β (**E**) in the supernatants were examined using ELISA. **F** The protein-ligand docking simulation of LDHA and doxorubicin. **G** LDH activity after doxorubicin treatment. **H** Transcriptomic expression of LDHA using single-cell RNA sequencing. *N* = 3. *P < 0.05, **P < 0.01.

Glycolysis is the metabolic pathway that converts glucose into pyruvate under anaerobic conditions. As glycolysis is accompanied by an increase in lactic acid production, we directly measured the extracellular lactic acid level. The results showed that doxorubicin increased extracellular lactic acid production (Fig. 5A).

We next addressed the possibility of doxorubicin increasing inflammatory cytokine expression by the increased synthesis of lactate. We supplemented lactate or inhibited lactate transporters with 2-cyano-3-(4-hydroxyphenyl)-2-propenoic acid (CHC) (Fig. 4C). Supplementing lactate elevated while blocking lactate transport attenuated the production of IL-6, TNF-α, and IL-1β in the several cultured macrophages and microglia (Fig. 5C-E).

To address the mechanism of lactic acid increase, we investigated the binding interactions of doxorubicin with amino acid residues of lactate dehydrogenase A (LDHA), catalyzing the conversion of pyruvate to lactate in the key steps of glycolysis, by protein-ligand docking simulation. Molecular docking simulation indicated that the maximum affinity of doxorubicin and LDHA is −8.329, and LDHA may interact with doxorubicin by forming four hydrogen bonds at the sites of amino acids ALA-97, ARG-98, ASN-137, and THR-247, according to the pharmacophore analysis (Fig. 5F). These binding sites confirm that these amino acid sequences may be key binding receptors in doxorubicin activity. Furthermore, LDH activity was consistently raised in macrophages and microglia after doxorubicin treatment (Fig. 5G). Additionally, A high level of LDHA transcriptomic expression was shown in myeloid cells of GBM patients in the data of single-cell RNA-seq (Fig. 5H).

Inhibiting LDHA by FX11 (Fig. 4C) reversed doxorubicin-induced increases in IL-6, TNF-α and IL-1β (Fig. 5C–E). As expected, LDHA knockdown (Fig. 5B) also blocked the increase of these cytokines mediated by doxorubicin (Fig. 5C–E), however, LDHA overexpression elevated the peoduction of these cytokines (Fig. 5C–E). These results suggest that doxorubicin-induced glycolysis to lactate may positively regulate the production of inflammatory cytokines.

**Fig. 6:**
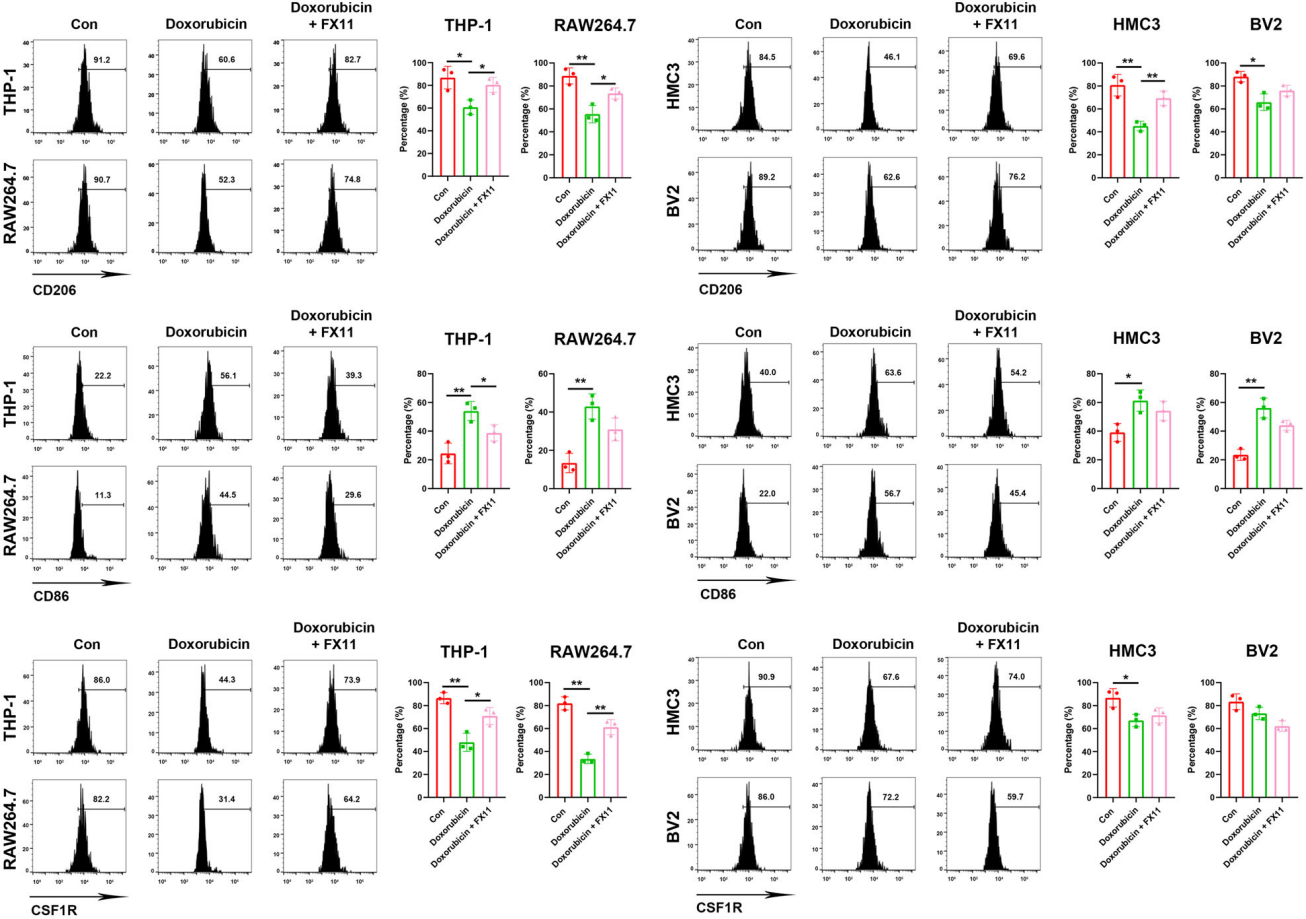
Inhibiting LDHA attenuates the immunosuppression reduction mediated by doxorubicin. Macrophages and microglia were supplemented with 20 ng/mL IL-4 and 20 ng/mL IL-13 for 48 h to induce M2-like polarization, then treated with 0.5 μM doxorubicin for 24 h, with or without 20 μM FX11 pretreatment for 3 h. The levels of CD206 (**A**), CSF1R (**B**), and CD86 (**C**) in cultured in vitro macrophages and microglia were then detected by flow cytometry. *N* = 3. *P < 0.05, **P < 0.01.

We next tested whether doxorubicin promotes immunosuppression through the LDHA/lactate axis. An M2-like polarized model was constructed using IL-4 treatment in macrophages and microglia cultured in vitro. Doxorubicin significantly decreased the expressions of immunosuppressive markers CD206 and CSF1R, while increasing the expression of immune-activated marker CD86. FX11 pretreatment partially reverted doxorubicin-induced changes in CD206, CSF1R, and CD86 in THP-1 and RAW264.7 cells. However, these markers were unchanged following FX11 pretreatment in doxorubicin-treated BV2 cells (Fig. 6). Collectively, these results suggest that doxorubicin attenuates immunosuppression and upregulates the expressions of inflammatory cytokines by regulating the LDHA/lactate axis.

## Discussion

**Fig. 7:**
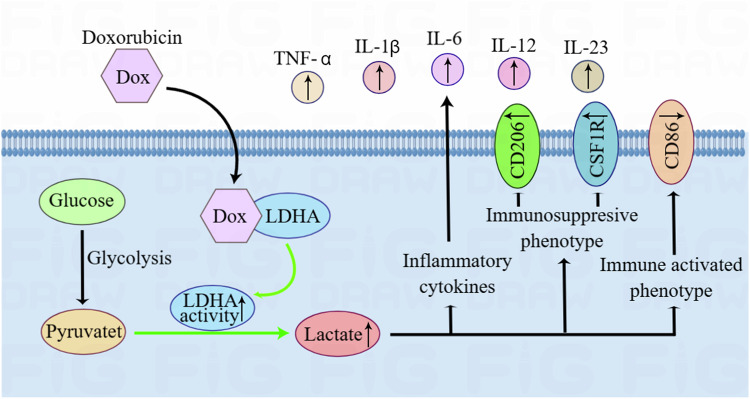
The mechanism of doxorubicin regulation on LDHA/lactate signaling axis in TAMs.

Recent research has shown that modifying cellular metabolic pathways is essential for immune cells [14]. Cancer cells show differences in growth and stress because they use substrates differently, leading to notable variations in how immune cells in the surrounding environment consume substrates, such as glucose [15]. Chemotherapy not only kills tumor cells, but also has a huge impact on immune cells. Here, we investigated the regulatory effect of doxorubicin on glucose metabolism of macrophages and microglia. We found that doxorubicin alleviates GBM immunosuppression by promoting glycolysis in macrophages and microglia. Mechanistically, doxorubicin binds LDHA to elevate lactic acid production, further promoting the release of inflammatory cytokines and inhibiting immunosuppressive phenotypes (Fig. 7).

Currently, a large number of studies have confirmed that doxorubicin affects cellular metabolism. The mechanism by which doxorubicin damages cell membrane is the alteration of sphingolipid metabolism [16]. Doxorubicin alters the lipid metabolism of liver cancer cells through reticulum stress response [17]. In addition, doxorubicin induces cardiotoxicity through NF-κB inflammatory signaling axis [18].

Glucose is an important nutrient that provides energy for cellular metabolic processes [19]. Once glucose is absorbed through the glucose transporter, it undergoes glycolysis to become pyruvate. If oxygen is present, pyruvate enters the TCA cycle. However, in the absence of oxygen, pyruvate is processed through glycolysis and is converted into lactate [20]. When inflammatory immune cells are activated, they switch from oxidative phosphorylation to aerobic glycolysis, leading to an increase in lactate, similar to the Warburg effect seen in cancer cells [21]. To initiate the inflammatory response, immune cells need to activate specific metabolic pathways [22]. Additionally, different types of immune cells depend on distinct metabolic processes and nutrient utilization [23]. Researches have demonstrated that the metabolism of macrophages can affect the production of inflammatory cytokines [24, 25]. We investigated the inflammatory response caused by the metabolic reprogramming of TAMs and found that the change in glucose metabolism in TAMs induced by doxorubicin resulted in inflammatory activation. It is one of the key mechanisms by which doxorubicin exerts anti-tumor immune effects.

Lactate, which is the final product of glycolysis, can influence the inflammatory response in different types of cells. During inflammation, immune cells generate and release large amounts of lactate [26]. Sodium lactate increases the LPS-induced production of MMP-1, IL-1β, and IL-6 in macrophage-like cells by triggering the NF-κB and MAPK signaling pathways [27]. Ablation of MYC reduces lactate production by controlling LDH activity and leads to an increase in inflammatory cytokines through the regulation of IRF4 [7].

The impact of lactate on inflammatory response is strongly linked to the cell phenotype and its metabolic condition. We demonstrated that doxorubicin inhibited the levels of the M2-like polarized molecule CD206 and the immunosuppressive phenotype marker CSF1R, as well as promoted the expression of the immune-activated phenotype marker CD86. Inhibiting lactate production with FX11 reversed the effect of doxorubicin on these molecules, indicating that doxorubicin regulates the immune phenotype of TAMs through lactate. A large body of research has also demonstrated the regulatory effect of lactate on the immune phenotype. LPS triggers the differentiation of macrophages into the M1 type, a proinflammatory form that produces ATP and releases lactate via aerobic glycolysis [28]. Additionally, blocking LDHA with FX11 decreases lactate production, the release of pro-inflammatory cytokines, iNOS levels, and COX2 expression in RAW264.7 macrophages stimulated with LPS [29]. MCT1, a lactate transporter, rises in response to inflammatory stimuli like LPS, TNF-α, or nitric oxide, leading to enhanced lactate uptake [30]. Taken together, these data suggest that lactate may be a metabolite involved in the regulation of the pro-inflammatory response in macrophages.

Lactic acid can not only provide tumor cells with energy but also act as a messenger molecule that promotes tumor growth and progression and protects tumor cells from immune cells and killing by radiation and chemotherapy [31]. We analyzed the possibility of doxorubicin binding to LDHA by molecular docking, which becomes the theoretical basis for doxorubicin effect on glucose metabolism. The binding efficiency and the molecular sites of their interaction will be further explored in the future.

Increased production of proinflammatory cytokines is also linked to histone lactylation [32]. Trained monocytes primarily use lactate instead of glucose as a substrate for the TCA cycle, and lactate metabolism is essential for trained immune cells. In addition to fueling the TCA cycle, both internally produced and externally supplied lactate promote trained immunity by influencing histone lactylation [33]. It can be seen that the upregulation of cytokines caused by increased lactate may be related to multiple factors, and we will continue to explore the mechanism in future study。

During inflammation, changes in cellular metabolism accompanied by increased extracellular acidification are well-established characteristics. The decrease in pH within inflamed tissues is primarily due to elevated lactate levels resulting from enhanced glycolysis. It has been shown that neutralizing the pH in a medium containing lactic acid enhances LPS-induced MMP-1 secretion, suggesting that the pH reduction caused by lactic acid may modulate pro-inflammatory responses [27]. Therefore, to eliminate the impact of proton increase, we used sodium lactate in this research and found that lactate upregulates the production of inflammatory cytokines.

Functionalized liposomes offer a more efficient method for delivering doxorubicin to tumors, overcoming major challenges related to crossing the blood-brain barrier. They extend circulation time and boost the accumulation of doxorubicin in tumor tissues. Liposomal delivery systems have greatly improved both the effectiveness and safety of doxorubicin compared to its free form. In this study, we used doxorubicin to investigate its anti-tumor mechanism in vivo. Due to limited ability of doxorubicin to penetrate the blood-brain barrier, we will conduct in vivo experiments using liposomal doxorubicin to better ensure its effective anti-tumor activity in GBM. These experiments aim to determine the optimal dosage of doxorubicin combined with TMZ for GBM treatment. Reducing the dosage of doxorubicin is intended to decrease its toxicity. Additionally, no significant toxicity was observed in our in vitro experiments at the doses used, however, these findings have not yet been validated in animal studies. In future research, we will evaluate the efficacy and toxicity of varying doses of doxorubicin against GBM in animal models, thereby establishing a theoretical foundation for further clinical investigations.

## Materials and methods

### Cell lines

GL261 cells were obtained from the American Type Culture Collection (ATCC) and cultured in high glucose DMEM. THP-1, RAW264.7, HMC3, and BV2 cells were purchased from the Cell Bank of the Chinese Academy of Sciences. THP-1 cells were cultured in RPMI 1640 medium, HMC3 cells were cultured in DMEM/F12 medium, while RAW264.7 and BV2 cells were cultured in high glucose DMEM. All media contained 10% fetal bovine serum (FBS) and 1% penicillin-streptomycin, and all cells were cultured at 37 °C in 5% CO_2_. THP-1 cells were grown in a six-well plate, and 100 ng/mL of phorbol ester (PMA) was used for 48 h to promote the differentiation into macrophages. For Cell polarization model establishment, THP-1, RAW264.7, HMC3, and BV2 cells were treated with 20 ng/mL IL-4 (PeproTech) and 20 ng/mL IL-13 (PeproTech) for 48 h to induce M2-like polarization.

### Animal experiment

All mice were handled in compliance with the guidelines set by the National Institutes of Health and Laboratory Animal Care, and the procedures were authorized by the Institutional Animal Care and Use Committee at Soochow University (No. 2021350). C57BL/6 J mice aged between 6 and 8 weeks were utilized for the experiments and were kept in specific pathogen-free environments. They had unrestricted access to food and water.

The mice were sedated using pentobarbital. A 5 μl cell suspension containing 1 × 10^5^ GL261 cells was then injected into the frontal lobe of the mouse brain through a stereotactic method to create an orthotopic GBM mouse model. The mice underwent RT with a dose of 2 Gy and were given 30 mg/kg of TMZ (Selleck) orally for five consecutive days, from day 10 to day 14. Additionally, doxorubicin was administered intraperitoneally at a dose of 4.5 mg/kg for three days, from day 10 to day 12. On day 20, the mice were euthanized by cervical dislocation, and the tumors were removed for IHC staining and flow cytometry detection (Fig. 1A).

For distribution detection, C57BL/6 J mice with an intracranial GL261-tumor were intraperitoneally injected doxorubicin linked to FITC. The tumor tissue of mice was removed 3 h after doxorubicin-FITC injection. Frozen sections were prepared, stained with DAPI, and observed under a fluorescence microscope.

### Immunohistochemistry (IHC) staining

Mouse tumor tissues were fixed in a 4% formalin solution, then dehydrated and embedded. 4 µm-thick paraffin sections were prepared. Antigen retrieval was performed using boiled citrate buffer, and endogenous peroxidases were deactivated with 0.3% H_2_O_2_ for 15 min. Next, nonspecific antigens were blocked using 5% bovine serum albumin. Primary antibodies, including Anti-Iba1 (Wako, Cat: 019-19741), anti-F4/80 (Abcam, Cat: ab111101), and anti-CD206 (Abcam, Cat: ab64693), were incubated overnight at 4 °C, followed by a 1 h incubation with the secondary antibody at room temperature. DAB Substrate (Abcam) and hematoxylin counterstaining were applied. Positive immune reactions were indicated by brown precipitates, which were observed under a light microscope.

### Preparation of single cell suspension and marker staining

Tumor tissues from the mice were dissociated for markers detection using flow cytometry, following the previously outlined method [34]. The cells were stained with anti-CD11b FITC (Invitrogen, Cat: 11-0112-82), anti-CD86 APC (Invitrogen, Cat: 17-0862-82), and anti-CD206 eFluor™ 450 (Invitrogen, Cat: 48-2061-82), anti-CSF1R PE (Invitrogen, Cat: 12-1152-82), and anti-CD45 PE-Cy7 (Invitrogen, Cat: 25-0451-82). LIVE/DEAD Fixable Blue Cell Stain Kit (Invitrogen) and anti-mouse IgG as a negative control were used. The staining cells were determined by a flow cytometer (BD Biosciences).

### Isolation of BMDMs

Bone marrow cells were extracted from the femurs and tibias of mice with tumors and then cultured in a 6-well plate at 37 °C with 5% CO_2_ using complete RPMI-1640 medium supplemented with 10% FBS. A final concentration of 20 ng/mL M-CSF was included in the medium for the culture of BMDMs. After 24 h of cell culture, the BMDMs and the supernatants were collected for cytokine analysis.

### siRNAs

LDHA siRNA was obtained from Genepharma Co., Ltd. Cells were transfected with either LDHA siRNA or a negative control siRNA in 6-well plates using Lipofectamine RNAiMAX, following the instructions provided by the manufacturer. The LDHA siRNA target sequences are as follow: Human LDHA-siRNA: 5′-CGAACTGGGCAGTATAAAC-3′; mouse Ldha-siRNA: 5’-GTTCCCAGTTAAGTCGTATAA-3’.

### Reverse transcription quantitative polymerase chain reaction (RT-PCR)

Total RNA was extracted from cultured cells or tumor tissues using TRIzol. cDNA was generated from 2 μg of RNA using a Reverse Transcription Kit (Applied Biosystems). Quantitative PCR was conducted according to manufacturer’s guidance. The primer sequences utilized for qPCR are provided below: murine Tnf, Forward: 5’-GTCAGGTTGCCTCTGTCTCA-3’, Reverse: 5’-TCAGGGAAGAGTCTGGAAAG-3’; murine Il1b, Forward: 5’-ACATCAGCACCTCACAAGCA-3’, Reverse: 5’-TTAGAAACAGTCCAGCCCATA-3’; murine Il6, Forward: 5’-AAGCCAGAGTCCTTCAGAGAGA-3’, Reverse: 5’-GGAAATTGGGGTAGGAAGGA-3’; murine Il12b, Forward: 5’-AGCACTCCCCATTCCTACTTCTCC-3’, Reverse: 5’-CACCCCTCCTCTGTCTCCTTCAT-3; murine Il23a, Forward: 5’-CACCAGCGGGACATATGAATCTAC-3’, Reverse: 5’-CTGGCTGTTGTCCTTGAGTCCTT-3’; Mouse Gapdh: Forward 5’-AAATGGTGAAGGTCGGTGTG-3’, Reverse: 5’-TGAAGGGGTCGTTGATGG-3’. human TNF, Forward: 5’-TCAATCGGCCCGACTATCTC-3’, Reverse: 5’-CAGGGCAATGATCCCAAAGT-3’; human IL6, Forward: 5′-CAATATTAGAGTCTCAACCCCCA-3′, Reverse: 5′-CCGTCGAGGATGTACCGAAT-3′; human IL1b, Forward: 5’-GCACGATGCACCTGTACGAT-3’, Reverse: 5’-AGACATCACCAAGCTTTTTTGCT-3’; human IL12B, Forward: 5’-ACCCTGACCATCCAAGTCAAA-3’, Reverse: 5′- TTGGCCTCGCATCTTAGAAAG-3’; human IL23A, Forward: 5’-CTCAGGGACAACAGTCAGTTC-3’, Reverse: 5′-ACAGGGCTATCAGGGAGCA-3’; human GAPDH, Forward: 5’-TGAAGGTCGGAGTCAACGGATT-3’, Reverse: 5’-CCTGGAAGATGGTGATGGGATT-3’. The relative expression of mRNA was adjusted based on GAPDH and shown as fold changes.

### Westen blot

Cells were lysed, and protein concentration was measured using the BCA protein assay kit (Beyotime). 20 μg of protein per lane were separated by 10% SDS-PAGE and then transferred onto nitrocellulose membranes. After blocking the membranes, they were incubated overnight at 4 °C with the primary anti-LDHA antibody (Cell Signaling Technology, Cat: 3582). The next day, the membranes were incubated with horseradish peroxidase-linked secondary antibodies at room temperature for 1 h. The immunoblots were detected using an enhanced chemiluminescence kit.

### Enzyme-linked immunosorbent assay (ELISA)

TNF-α, IL-1β, and IL-6 levels in the supernatants of mouse BMDMs, tumor tissues, in vitro cultured cells were detected by Mouse or Human ELISA Kits (Abclonal), and respectively strictly according to the manufacturer’s instructions.

### Flow cytometry

Cells of 1 × 10^6^ cultured in vitro were gathered into a 15 ml tube. PE conjugated anti-CD206 antibody (eBioscience™, Cat: 12-2069-42 and 12-2061-82), PE conjugated anti-CD115 antibody (eBioscience™, Cat: 12-1159-42 and 12-1152-82), and PE conjugated anti-CD86 antibody (eBioscience™, Cat: 12-0869-42 and 12-0862-82) were used for cell staining at room temperature for 10 min in the dark, followed by analysis with a flow cytometer (BD Biosciences).

### Measurement of cellular ATP concentrations

Cells were seeded in a 96-well clear bottom plate with 100 μL of medium and allowed to adhere overnight. The Luminescent ATP Detection Assay Kit (Beyotime) was utilized to assess cellular ATP levels following treatment with doxorubicin, with or without 2-DG. In brief, 50 μL of detergent was added to the existing 150 μL in each well. The plate was gently rocked for 5 min at room temperature. Subsequently, 50 μL of substrate from the kit was introduced, and the plate was rocked again for another 5 min in the dark. After that, the plate was left in the dark for 10 min. Luminescence from the wells was then measured using a luminometer (Thermo Fisher).

### Measurement of glucose uptake

A fluorescent glucose analog, 6-(N-(7-nitrobenz-2-oxa-1,3-diazol-4-yl) amino)-2-deoxy-glucose (6-NBDG, Molecular Probes), was utilized to assess glucose uptake. Cells were treated with 1 nM insulin for 10 min, after which the medium was replaced with glucose-free DMEM, and 300 μM 6-NBDG was added to all cells for 45 minutes at 37 °C. The cells were then thoroughly washed to eliminate any external fluorescent glucose analog, and the fluorescence intensity of 6-NBDG was measured at a wavelength of 540 nm (with an excitation wavelength of 465 nm) using flow cytometry.

### Assay of glucose consumption, lactate production, and LDHA activity

Concentrations of glucose and lactate in the supernatants and cellular LDH activity were measured using Glucose kit, Lactic acid assay kit, and Lactate dehydrogenase assay kit (Nanjing Jiancheng Bioengineering Institute) by colorimetric assay according to the manufacturer’s instructions. The concentrations of glucose and lactate obtained were subtracted from the initial values of the cell culture media to determine the relative rates of glucose consumption and lactate production. These concentration values were adjusted based on the number of cells.

### CCK-8 assay

Cell proliferation was detected by a cell counting kit (CCK-8). The cells were seeded in 96-well plates at a density of 2000 cells/well. Following indicated treatment, CCK-8 was added with 10 μl per well and incubated for 2 h. The absorbance at 450 nm was measured using a microplate reader.

### Molecular docking

The PDB files of doxorubicin and LDHA protein were downloaded from the PDB database (https://www.rcsb.org.). Molecular docking obtaining the affinity and result visualization were performed using autodock software (https://autodock.scripps.edu/) and online molecular docking website (https://www.easy2md.com/).

### Statistical analysis

All data in vitro reflect results from at least three independent experiments. Groups were assigned randomly. Statistical evaluations were conducted using GraphPad Prism software. Error bars indicate ±standard error of the mean (S.E.M.) as calculated by Prism. The specific statistical tests employed included paired and unpaired Student’s t-tests and two-way ANOVA, as detailed in the figure legends. A p-value of less than 0.05 was deemed statistically significant.

## Supplementary information

Supplementary Figures

Full and Uncropped Western Blots

## Data Availability

All relevant data can be obtained from the corresponding author upon request.
