# Supplementary material for: Doxorubicin promotes the production of inflammatory cytokines in tumor-associated macrophages through activating lactate dehydrogenase A
Source: Cell Death Discov. 2026 Mar 31;12:208. doi: 10.1038/s41420-026-03014-0 (PMC13158295; doi:10.1038/s41420-026-03014-0)
Supplement: Supplementary file 1 — Supplementary Figures [file 41420_2026_3014_MOESM1_ESM.pdf]

Supplementary Figures

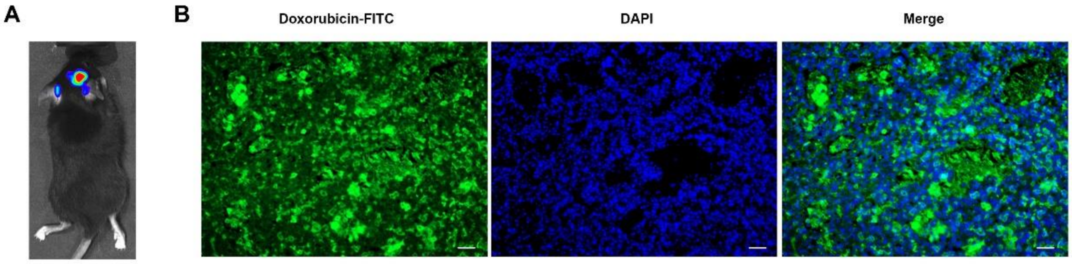

Supplementary Figure S1. The distribution of doxorubicin-FITC in the tumor of mice.

(A) GL261 cells were transfected with luciferase lentivirus, then intracranially injected into C57BL/6J mouse. Intracranial tumor formation was confirmed through live imaging. (B) The doxorubicin was linked to FITC. The tumor tissue of mice was removed 3 hours after doxorubicin-FITC injection. Frozen sections were prepared and observed under a fluorescence microscope. Scale bars correspond to 100 μm.

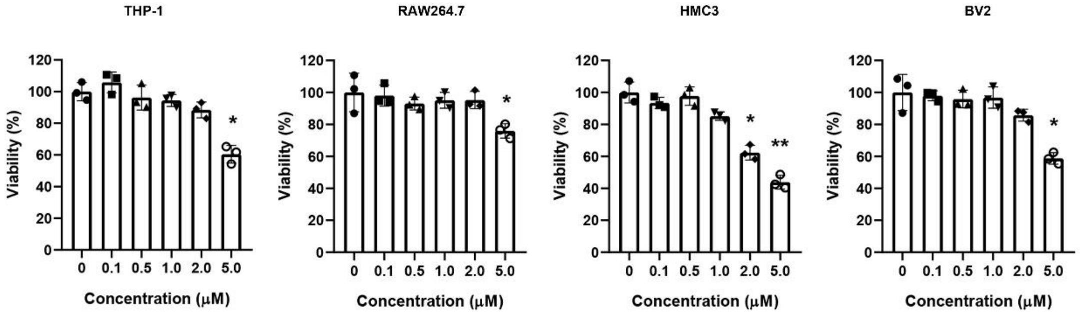

Supplementary Figure S2. The viability of macrophages and microglia after different dosage of doxorubicin treatment for 24 hours.

The percentage of viability is calculated according to the absorbance value of the indicated concentration divided by that of 0 μM. $*P < 0.05$, $**P < 0.01$ vs. 0 μM.

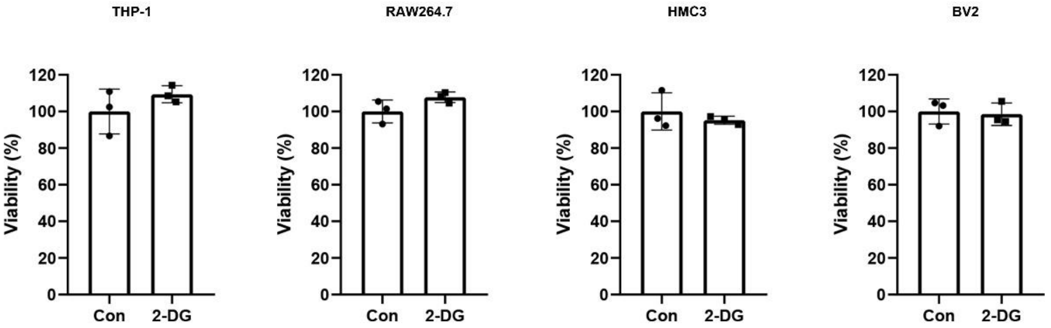

Supplementary Figure S3. The viability of macrophages and microglia after 2-DG treatment.

Cells were treated with 5 mM 2-DG for 0.5 h, then the supernatants were discarded and cells were cultured in common medium for 24 hours. Cell viability was detected by CCK-8. The percentage of viability is shown as the absorbance value of the indicated dosage divided by that of 0 μM.
