# Supplementary material for: Doxorubicin promotes the production of inflammatory cytokines in tumor-associated macrophages through activating lactate dehydrogenase A
Source: Cell Death Discov. 2026 Mar 31;12:208. doi: 10.1038/s41420-026-03014-0 (PMC13158295; doi:10.1038/s41420-026-03014-0)
Supplement: Supplementary file 2 — Full and Uncropped Western Blots [file 41420_2026_3014_MOESM2_ESM.pdf]

Supplementary Imagines in Western blot

Full unedited gel for Figure 5B

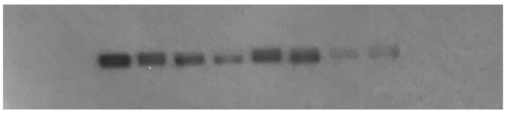

Lane 2, 3, 4 are control, scramble transfection, and siRNA-LDHA transfection in THP-1 cells.

Lane 5, 6, 7 are control, scramble transfection, and siRNA-LDHA transfection in HMC3 cells.

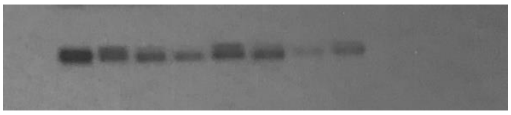

Lane 2, 3, 4 are control, scramble transfection, and siRNA-LDHA transfection in RAW264.7 cells.
Lane 5, 6, 7 are control, scramble transfection, and siRNA-LDHA transfection in BN2 cells.

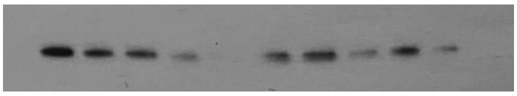

Lane 1 and 2 are scramble transfection and siRNA-LDHA transfection in THP-1 cells.
Lane 3 and 4 are scramble transfection and siRNA-LDHA transfection in HMC3 cells.
Lane 7 and 8 are scramble transfection and siRNA-LDHA transfection in RAW264.7 cells.
Lane 9 and 10 are scramble transfection and siRNA-LDHA transfection in BV2 cells.

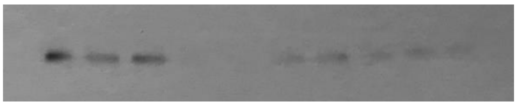

Lane 1 and 2 are scramble transfection and siRNA-LDHA transfection in THP-1 cells.
Lane 3 and 4 are scramble transfection and siRNA-LDHA transfection in HMC3 cells.
Lane 7 and 8 are scramble transfection and siRNA-LDHA transfection in RAW264.7 cells.
Lane 9 and 10 are scramble transfection and siRNA-LDHA transfection in BV2 cells.

Antibody: Anti-LDHA antibody (Cell Signaling Technology, Cat: 3582)
